# Deep convolutional neural networks outperform vanilla machine learning when predicting language outcomes after stroke

**DOI:** 10.1016/j.nicl.2025.103880

**Published:** 2025-09-29

**Authors:** Thomas M.H. Hope, Howard Bowman, Alex P. Leff, Cathy J. Price

**Affiliations:** a Department of Imaging Neuroscience, Institute of Neurology, University College London, 12 Queen Square, London WC1N 3AR, the United Kingdom of Great Britain and Northern Ireland; b School of Psychology University of Birmingham Edgbaston Birmingham B15 2TT the United Kingdom of Great Britain and Northern Ireland; c Department of Psychological and Social Sciences, John Cabot University, Via della Lungara 233, 00165, Rome, Italy

**Keywords:** Stroke, Language, Cognition, Machine learning, Lesions, Deep learning

## Abstract

**Background:**

Current medicine cannot confidently predict patients’ language skills after stroke. In recent years, researchers have sought to bridge this gap with machine learning. These models appear to benefit from access to features describing where and how much brain damage these patients have suffered. Given the very high dimensionality of structural brain imaging data, those brain lesion features are typically post-processed from the images themselves into tabular features. With the introduction of deep Convolutional Neural Networks (CNN), which appear to be much more robust to high dimensional data, it is natural to hope that much of this image post-processing might be unnecessary. But prior attempts to demonstrate this (in the area of post-stroke prognostics) have so far yielded only equivocal results – perhaps because the datasets that those studies could deploy were too small to properly constrain CNNs, which are famously ‘data-hungry’.

**Methods:**

The study draws on a much larger dataset than has been employed in previous work like this, referring to patients whose language outcomes were assessed once during the chronic phase post-stroke, on or around the same days as they underwent high resolution MRI brain scans. Following the model of our own and others’ past work, we use state of the art ‘vanilla’ machine learning models (boosted ensembles) to predict a variety of language and cognitive outcomes scores. These models employ both demographic variables and features derived from the brain imaging data, which represent where brain damage has occurred. These are our baseline models. Next, we use deep CNNs to predict the same language scores for the same patients, drawing on both the demographic variables, and post-processed brain lesion images: i.e., multi-input models with one input for tabular features and another for 3-dimensional images. We compare the models using 5 × 2-fold cross-validation, with consistent folds.

**Results:**

The CNN models consistently outperform the vanilla machine learning models, in this domain.

**Conclusions:**

Deep CNNs offer state of the art performance when predicting language outcomes after stroke, outperforming vanilla machine learning and obviating the need to post-process lesion images into lesion features.

## Highlights

• Recent research used machine learning to predict language outcomes after stroke.
• We show that deep learning can outperform a strong baseline from that literature.
• This advantage was consistent across many outcome scores.

## 1 Introduction

Stroke is the leading cause of overall disease burden in the world GBD Nervous System Disorders Collaborators (2024). Patients who survive the initial insult are often left with cognitive impairments, and want to know whether and when the might recover. During the last decade (or so), researchers have sought to answer these questions using machine learning (e.g., (Hope et al., 2013, Del Gaizo et al., 2017, Pustina et al., 2017, Hope et al., 2018)). These machine learning models benefit from access to features describing where and how much lesion damage each patient has suffered (Hope et al., 2013, Hope et al., 2015, Hope et al., 2018, Hope et al., 2018, Loughnan et al., 2019), but whole-brain lesion images are high dimensional, and classical machine learning models are not robust to very high dimensional predictor data. The popular response has been to re-encode those lesion images to reduce their dimensionality while preserving as much predictive power as possible (Yourganov et al., 2016, Hope et al., 2018, Nieto-Castañón, 2022, Talozzi et al., 2023). Deep learning should be a natural alternative, because these models have proved to be robust to very high dimensional input feature spaces (Teghipco et al., 2023), so could potentially be used to circumvent the encoding process. But it has proved to be surprisingly difficult to demonstrate a definite advantage for deep learning over classical machine learning when predicting outcomes after stroke: this is our focus in the current work.

A recent study by Teghipco and colleagues (Teghipco et al., 2023) provides an up to date review of the ways in which deep learning is being used in this space. We will not repeat that effort here, save to re-state those authors’ conclusions: these methods have so far found rather more purchase in brain image processing applications, such as image segmentation (Ronneberger et al., 2015), than they have in outcome prediction applications. At the time of writing, Teghipco and colleagues’ own study was the only one (that we know of) to successfully demonstrate an advantage for deep learning when predicting language outcomes after stroke. The authors’ dependent variable was the Aphasia Quotient (AQ), an overall score summarising language task scores from the Western Aphasia Battery (WAB). They trained their model to use patients’ brain images (normalised and warped into standard space, with lesions manually drawn on) to classify whether or not the patients were ‘severely aphasic’ at test time. Their model, whose architecture was based on the VGG network (Simonyan and Zisserman 2015), significantly outperformed baseline, classical machine learning models employing principal components of the original brain images.

More recently, we produced another demonstration, with a larger sample (White et al., 2024). The outcome variable in our study was scores in a task requiring participants to describe the scene depicted in a picture. This task is perhaps the hardest component of the Comprehensive Aphasia Test (CAT) (Swinburn et al., 2004), in the sense that participants who score well must have relatively preserved or recovered function in a variety of cognitive domains. We divided that scale into ‘normal’ versus ‘aphasic’ ranges; participants scoring in the latter range are below the fifth percentile of scores assigned to a separate population of stroke patients. One innovation in this case was the use of data fusion to augment the brain images with features representing non-imaging predictors (such as age at stroke onset), which have proved to convey prognostic value in past studies (Hope et al., 2013, Seghier et al., 2016, Hope et al., 2018, Loughnan et al., 2019, White et al., 2024). So long as both baseline and deep learning models have access to the same information, the comparison between them should (or can) be fair. But while a constrained comparison – omitting some information that is probably relevant to problem – can make the case that one model uses shared information better than another, it cannot directly address the practical question of whether we should use one model or another to actually predict real prognoses in practice. Our data fusion method allowed us to compare like with like, without removing potentially important information from either model.

We also recently submitted another study using deep learning methods with a subset of the current study’s sample. The focus of this work is a ‘learning curve’ analysis, with just two outcome scores: i.e., the aim was to explore how learning performance changes as the training sample size grows. Like the two studies discussed so far, this study employed cut-off thresholds to convert the outcome scores into binary classes (‘recovered’ versus ‘impaired’). There is nothing wrong with this shift in principle, not least because the resultant classes might be more interpretable, and thus clinically relevant, than the original scores (Cheng et al., 2020, Cheng et al., 2023). But the regression problem is arguably both more faithful to the original data and more general, in the sense that continuous predictions can be re-encoded into whatever classes are deemed to be useful. The re-encoding also divorces these studies from much of the prior literature in this field, which treats this problem as a regression (Hope et al., 2013, Zhang et al., 2014, Hope et al., 2015, Pani et al., 2016, Yourganov et al., 2016, Del Gaizo et al., 2017, Hope et al., 2017, Ramsey et al., 2017, Halai et al., 2018, Hope et al., 2018, Pustina et al., 2018, Roohani et al., 2018, Loughnan et al., 2019, Halai et al., 2020, Hope et al., 2021, Akkad et al., 2023, Talozzi et al., 2023, White et al., 2024, Tilwani et al., 2025). This means that none of the classification studies can directly compare their model to a strong baseline, justified by that prior literature. This is important because deep learning appears to be better suited to classification than it is to regression (Liu et al., 2021, Foumani et al., 2024). Apparently strong classification results might therefore not guarantee that this technology will solve the traditional regression problem especially well.

At least as far as we know, the only prior attempt to demonstrate the utility of deep learning when making point predictions of language outcomes after stroke, by direct comparison to a strong baseline model, was by Chauhan and colleagues (Chauhan et al. 2019). Like the study by Teghipco and colleagues, these authors restricted their analysis to imaging-related prognostic factors only. This study found no consistent benefit of using CNNs over baseline, classical machine learning models. However, the authors did observe that features extracted from the images, via a CNN, conveyed useful prognostic power over and above those encoded by principal components of the same images. And because this unique information emerged only when most or all of the sample (n = 132) was used for training, the authors concluded that more consistent benefits might emerge with still larger samples than they could bring to bear.

Here, we test that prediction by using CNNs to predict outcome scores (i.e., with regression models) with a very large sample of patients, across a wide range of cognitive and language outcomes scores. Our baseline model is selected based on prior work aiming to predict the same outcomes scores in a subset of the sample employed in this study (Hope et al., 2018). And we also adopt a multi-input architecture so that non-imaging (tabular) features can be included. Our results suggest that the deep learning architecture is pareto optimal relative to our baseline model: i.e., often better, and never worse.

## 2 Methods

### 2.1 Data

Our patient data are drawn from the Predicting Language Outcome and Recovery After Stroke (PLORAS) dataset (Seghier et al., 2016), which associates more than 1,500 S survivors with: (a) clinical and demographic data; (b) high resolution, T1-weighted structural MRI; and (c) scores from the Comprehensive Aphasia Test (CAT) (Swinburn et al., 2004): a standardised battery of behavioural tasks, designed to assess the severity of participants’ language and cognitive impairments. Data were acquired primarily but not exclusively in the chronic phase, ranging from 2 months to > 10 years post-stroke. Patients were excluded from these analyses only if they had no relevant language assessment scores to predict. Where patients had more than one record in the database (indicating a repeat visit and assessment), only the first was used.

### 2.2 Language assessment scores

The CAT defines 34 task scores, including 29 that refer to language skills, and 5 that refer to non-linguistic, general cognitive skills (Swinburn et al., 2004). Since our interest is general and methodological rather than specific to any individual outcome score, we implemented 34 model comparisons for pairs of models predicting each of the 34 scores. The scores capture patients’ skills in speaking, comprehension, reading and writing, as well as visual attention, memory and arithmetic: collectively, they provide a reasonably comprehensive profile of each patient’s cognitive skills after stroke. Detailed descriptions of these scores, and the tasks from which each score is derived, are provided in the manual for the CAT (Swinburn et al., 2004). Scores are represented as t-scores, defined relative to a distribution of scores for each task, acquired from 236 people with aphasia. Lower t-scores imply worse task performance, and therefore more severe impairment in that task. Our models attempt to predict those t-scores directly: i.e., they are regression models.

### 2.3 Brain imaging data

Our T1-weighted MRI scans were acquired using a variety of Siemens scanners since 2013 – typically but not exclusively on the same day as, or within a few days of, the conduct of the behavioural assessment. All scans were processed using the Automatic Lesion Identification (ALI) toolbox (Seghier et al. 2008), which is an elaboration of the popular Unified Segmentation algorithm (Ashburner and Friston 2005), adapted for use in the damaged brain. The ALI toolbox derives continuous lesion evidence at the voxel level by comparing each participant’s scan to a distribution of reference scans, acquired on the same scanners from neurologically normal controls. The result is a whole-brain continuous lesion image, in standard Montreal Neurological Institute space.

### 2.4 Baseline models

Our baseline models are boosted decision trees, which have consistently produced state-of-the-art performance on a wide variety of machine learning problems in the past (Nielsen 2016), and which were consistently effective in our own past work predicting the same outcome variables for a subset of the current sample (Hope et al., 2018). We implement the model using the python XGBoost library (Chen and Guestrin 2016).

These models are driven by tabular features representing both basic demographic information (time post-stroke at assessment, age at stroke onset, sex assigned at birth, pre-stroke handedness, and bilingualism status) and lesion information. The lesion information includes both the extent of lesion damage (binary lesion volume in each of the two hemispheres), and its location. To capture the latter type of information, we re-encode the lesion images as the mean lesion signal in each of a series of 398 regions of interest. Region masks are drawn from 4 publicly available atlases: 2 focused on grey matter (Tzourio-Mazoyer et al., 2002, Eickhoff et al., 2005) and 2 focused on white matter (Hua et al., 2008, Oishi, 2011). Taken together, these masks allow us to represent lesion location in a flexible manner using hundreds of variables, rather than hundreds of thousands of lesion images voxels. This approach mirrors that of most prior, analogous work in this field: e.g., (Zhu et al., 2010, Hope et al., 2013, Wang et al., 2013, Gillebert et al., 2014, Mah et al., 2014, Kuceyeski et al., 2016)).

### 2.5 Deep computer vision models

**Fig. 1:**
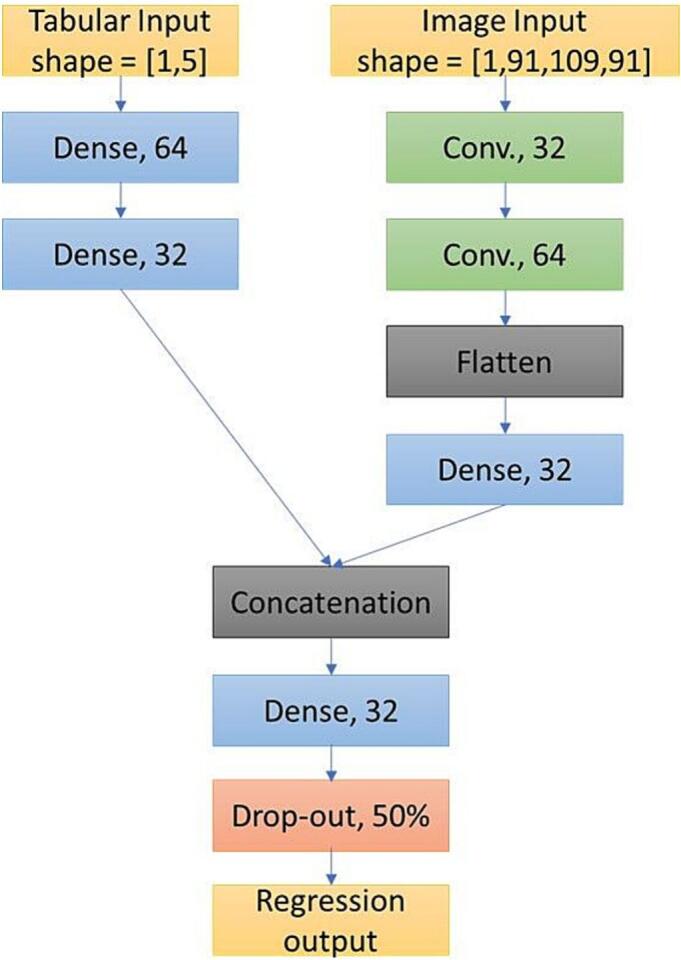
A schematic illustration of our deep learning model. A regression model with two inputs: one for 5 tabular features (time post-stroke at assessment, age at stroke onset, pre-stroke handedness, sex assigned at birth, and bilingualism status), and the other for continuous lesion images of size 91 × 109 × 91 2 mm^3^ voxels. ‘Dense’ layers are fully connected, feedforward layers. ‘Conv.’ layers are convolutional layers. All other hyperparameters are left at their defaults, as defined by the Tensorflow library with a Keras backend. All activation functions were rectified linear.

Our deep learning models are convolutional neural networks (CNNs), which have matched or exceeded more classical machine learning approaches on a wide variety of computer vision benchmarks (Krizhevsky et al., 2012). CNNs are perhaps best known for 2-dimensional image processing problems, so when using these models to predict PLORAS patients’ outcomes in the past, some of us have preferred to convert the 3-dimensional brain images into 2-dimensional ‘stitched’ images: i.e., a mosaic of axial brain slices (Roohani et al., 2018, White et al., 2024). But while this conversion has proved effective, we use the images in their original, 3-dimensional form in this work. A schematic of the model is shown in Fig. 1. We use the Adam learning method, and train for 10 epochs, reporting prediction loss (Mean Squared Error: MSE) on the test set. The batch size for CNN training is set to 64, because larger batch sizes caused occasional memory overflow errors.

### 2.6 Model comparison

Our sample is one of the largest of its kind in the world, but it is still small by the standards of the machine learning community. We use what we believe is currently the most popular method for model comparison with small samples: a *t*-test for paired samples on test set losses (from the baseline and network models, respectively) derived from 5 repetitions of a split-half cross-validation (Dietterich 1998). Having selected the sample, each iteration of the method starts by dividing that sample into two random subsamples. We then train each model (i.e., the baseline model and the CNN) using one subsample and use them to predict outcomes for the other subsample, before swapping the training and test samples and repeating the same procedure. Repeating the whole process 5 times, with 5 random partitions of the data, gives us a total of 10 loss metrics (mean squared errors) for both the baseline model and the CNN. Since the data partitions are constant across the models, we can compare those measures using a *t*-test for paired samples. The better model should have smaller magnitude prediction errors, and since we ran this analysis specifically to measure any advantage for the CNN, we use one-tailed t-tests to compare the CNN to the baseline model. The whole procedure is then repeated 34 for times, for each of the 34 outcomes scores that we consider.

## 3 Results

### 3.1 The sample

A total of 1,367 patients met the inclusion criteria for the study, including 458 women and 909 men. Where patients had visited us twice, and thus had more than one record in the PLORAS database, we included only the first. 1,195 were right handed pre-stroke, with the remainder either left handed or ambidextrous. 1,223 were native English speakers. The average age at stroke was 56 years ± 13.3 years, and the average time post-stroke at assessment was 46.3 months ± 56.8 months. The average lesion volume was 64.0 cm^3^ ± 82.7 cm^3^. Some patients had missing outcomes data for some of the 34 tasks, and were therefore omitted from the analysis, leaving a total sample size of 1,259.

### 3.2 Model comparison

Table 1 reports the results of model comparisons across all 34 task scores defined by the CAT. The CNN significantly outperforms the baseline model in 30/34 tasks (p < 0.05). The same tasks also exceed a 5 % threshold for significance derived via permutation thresholding (threshold t = 1.83) with 10,000 permutations. In 18/34 tasks, the difference also meets the Bonferroni-corrected 5 % threshold. The baseline model never significantly (or even numerically) outperforms the CNN. In this sense, at least with these data, our CNN appears to be pareto optimal relative to the baseline model.

**Table 1:** Model comparison statistics for models predicting all 34 task scores defined by the Comprehension Aphasia Test, derived via 5 times 2-fold cross-validation. Mean Squared Errors (MSE) for the CNN were significantly smaller than those for the baseline model in 30/34 tasks (p < 0.05), and numerically smaller in the other 4 tasks. The table includes the number of participants with outcomes data for each task (N), the number assessed as impaired on that task (I), and the average correlation between predicted scores and empirical scores (R).

TASK					BASELINE		CNN MSE	
	N	I	P	T	MSE	R	MSE	R
Line bisection	1346	97	<0.001	7.48	1.10	0.16	0.98	0.20
Semantic memory	1367	139	<0.001	6.88	1.05	0.24	0.96	0.28
Fluency	1366	359	0.012	2.69	0.55	0.68	0.52	0.70
Recognition memory	1364	166	<0.001	11.77	1.16	0.09	1.01	0.14
Gesturing object use	1362	207	<0.001	7.52	1.00	0.30	0.91	0.33
Mental arithmetic	1357	41	0.002	3.83	1.03	0.27	0.92	0.32
Memory (total)	1362	166	0.001	4.27	1.04	0.21	0.97	0.24
Comprehension of spoken words	1366	250	<0.001	4.96	0.87	0.41	0.78	0.47
Comprehension of spoken sentences	1361	560	0.016	2.53	0.66	0.62	0.62	0.65
Comprehension of spoken paragraphs	1354	162	<0.001	9.33	0.99	0.30	0.86	0.32
Comprehension of spoken language (total)	1352	428	<0.001	6.87	0.66	0.60	0.63	0.63
Comprehension of written words	1364	407	<0.001	5.14	0.84	0.45	0.75	0.49
Comprehension of written sentences	1359	433	0.014	2.62	0.67	0.59	0.62	0.63
Comprehension of written language (total)	1358	527	0.004	3.45	0.65	0.59	0.62	0.63
Repeating words	1360	448	0.007	3.06	0.69	0.57	0.67	0.59
Repeating complex words	1358	371	<0.001	6.13	0.67	0.59	0.62	0.61
Repeating non-words	1359	365	<0.001	7.11	0.79	0.50	0.71	0.54
Digit span	1360	375	0.086	1.48	0.61	0.62	0.61	0.65
Repeating sentences	1357	421	0.001	4.50	0.52	0.70	0.47	0.74
Repetition (total)	1354	677	0.038	2.01	0.56	0.67	0.54	0.68
Object naming	1360	522	0.025	2.26	0.59	0.66	0.56	0.67
Action naming	1356	637	<0.001	8.14	0.62	0.63	0.57	0.66
Naming (total)	1350	490	0.237	0.75	0.49	0.72	0.47	0.73
Scene description	1346	585	0.001	4.63	0.55	0.67	0.52	0.69
Reading words	1353	539	0.001	4.26	0.63	0.61	0.60	0.65
Reading complex words	1348	443	0.023	2.30	0.62	0.63	0.60	0.65
Reading function words	1351	130	<0.001	7.23	0.82	0.49	0.72	0.52
Reading non-words	1351	460	<0.001	5.01	0.61	0.63	0.59	0.65
Reading (total)	1348	481	0.058	1.74	0.58	0.66	0.55	0.68
Writing (copying)	1336	145	0.001	4.62	0.96	0.31	0.89	0.36
Written object naming	1339	260	0.003	3.61	0.80	0.49	0.74	0.53
Writing to dictation	1337	420	0.015	2.59	0.68	0.58	0.65	0.60
Writing (total)	1324	383	0.073	1.59	0.68	0.57	0.67	0.60
Written scene description	1310	533	0.002	3.78	0.63	0.62	0.58	0.66

Table 1 also includes the correlations between predicted and empirical scores for each model and task. These figures are provided to illustrate the models’ relative performance in what might be more meaningful terms than the MSE values. However, these measures should be interpreted with caution, because our model comparison method is designed around MSE rather than correlations. Specifically, we use 2-fold cross-validation to minimise the overlap between training sets for each fold, thereby recovering what is thought to be enough independence to use paired statistics for the model comparisons (Dietterich 1998). The cost, is smaller training sets, and therefore potentially also weaker predictions. Building on prior results (e.g., (Shao 1993)), we have preferred 10-fold cross-validation in work where the metric of interest is the correlation between predicted and empirical scores.

## 4 Discussion

When predicting the outcomes of a large sample of stroke patients, we have demonstrated a statistically significant advantage for deep CNNs over state of the art, classical machine learning. The advantage was consistent when predicting a wide range of outcomes scores defined by a popular, standardised battery of tests of these patients’ language and cognitive skills. Indeed, our baseline model under-performed (at least numerically), relative to the CNN, on every task that we employed. As far as we know, this is the first time that this advantage has been demonstrated in this domain.

We speculate that this advantage is a function of the different ways in which each model encodes lesion images. Our baseline models encode them as a series of 398 lesion load variables, an encoding that necessarily trades resolution for dimensionality reduction. Crucially, that dimensionality reduction operates independently of the outcome variable that we want to predict, so might obscure information that is relevant to those predictions. By contrast, the lesion images are fed into the CNN in their native form, with all voxel values preserved. The model then learns to reduce those images’ dimensionality at the same time as it learns to predict the relevant output variable, so those two processes are integrated during the learning.

Given the large size of our sample, relative to standards of the field, this result is consistent with the prediction made by Chauhan and colleagues (Chauhan et al., 2019). However, our results also suggest another explanation for their null result, because 3 of the 4 scores for which no significant deep learning advantage was found (p >= 0.05; see Table 1) are what we call ‘total’ scores: scores which effectively summarise other scores (for naming, reading and writing). These total scores are in a sense more similar to the summary score used by Chauhan and colleagues – the first principal component of outcomes scores. It might be that higher resolution lesion information is relatively less useful when predicting summary-level outcomes than it is when predicting tasks that tap more specific cognitive functions. Some support for this claim also accrues from prior results: e.g., (Thye and Mirman, 2018, Sperber et al., 2023). Of course, our data also differ from that reported by Chauhan and colleagues in other ways that might explain the discrepancy between our results and theirs, including: (a) the timing of scan acquisition (ours are chronic whereas theirs were acute); and (b) the type of lesion information employed (theirs are binary lesion images whereas ours represent whole-brain structural abnormality).

This work could be extended in several ways. First, it seems wasteful to train so many separate models when the dependent variables are likely so correlated – as we know and expect to be the case when task batteries include tasks engaging related functions (such as reading single words and reading complex words). It should be possible to take advantage of that dependent variable structure, using multi-output learning: i.e., training a single model to predict all CAT task scores together. This is a focus of ongoing work.

Second, the data employed in this study are still somewhat divorced from the target application for this work. Our over-arching interest is naturally in predicting later (chronic phase) outcomes from patient data (scans and tabular variables) acquired acutely. However, that longitudinal data is challenging to acquire at scale, and challenging to use because of the complexities and inconsistencies inherent in data acquired routinely for clinical use, especially when the patient’s life it at stake (as it often is soon after stroke). Therefore, like many others in the field (Talozzi et al., 2023, Teghipco et al., 2023, White et al., 2024), we approach this issue using cross-sectional data: i.e., using predictors derived from patient data acquired at the same time as language outcomes scores were acquired (typically in the chronic phase post-stroke). In this sense, what we are doing cannot be described as ‘predicting recovery’ after stroke. Instead, we are ‘predicting outcomes’, because we are generalising models trained with patient data to new patients: i.e., patients whose data (predictors and outcomes) were not seen during training.

The two types of problem are similar in the sense that both could in principle employ (very) similar predictors and outcome measures. We might approach those data in the same way in both problems, but models trained for one problem probably won’t generalise to the other without at least some data augmentation. We explored this in an earlier study, in which we generalised a model trained on a subset of the data used here (chronic predictors and outcomes) to patients whose predictors were acquired acutely (with outcome scores acquired later, in the chronic phase) (Loughnan et al., 2019). We found that the model performed less well in the longitudinal sample than in the cross-sectional sample, with the latter assessed via cross-validation. We speculated that the difference might have been a function of a distribution shift caused by the growth of patients’ lesions over time post-stroke: i.e., lesions that appeared small in the acute images might be larger in the chronic images used to train the models. Support for that speculation accrued from applying a simple growth function to the acute lesion images, which significantly improved performance for those longitudinal patients. The more elegant solution is of course to train new models with longitudinal data only. We see no reason to think that the advantage that we have observed here, for our deep learning model versus the baseline model, would disappear in that case.

Nevertheless, at least in this specific domain, our results suggest that our CNN is effective, relative to a state-of-the-art baseline. This advantage is remarkably consistent, suggesting that the CNN was pareto optimal relative to our baseline: i.e., often (significantly) better and never worse. Naturally, results like this are always contingent on the models actually employed. Some better, classical machine learning model might yet extinguish the advantage we see here – just as some more optimised neural network architecture might yet increase it. But given the data and methods that were available to us here, the evidence is as clear as it can be: our CNN seems the better way to tackle this problem.

## CRediT authorship contribution statement

**Thomas M.H. Hope:** Writing – review & editing, Writing – original draft, Validation, Software, Methodology, Formal analysis, Conceptualization. **Howard Bowman:** Writing – review & editing, Methodology, Conceptualization. **Alex P. Leff:** Writing – review & editing, Validation, Resources, Methodology, Investigation. **Cathy J. Price:** Writing – review & editing, Validation, Supervision, Project administration, Investigation, Funding acquisition, Data curation, Conceptualization.

## Declaration of Competing Interest

The authors declare that they have no known competing financial interests or personal relationships that could have appeared to influence the work reported in this paper.

## Data Availability

Data can be shared on the conclusion of a data sharing agreement with UCL
